# Normative CLEFT-Q Data From the General Dutch Population

**DOI:** 10.1097/SCS.0000000000010882

**Published:** 2024-11-21

**Authors:** Philip A.J. van der Goes, Victor L. Van Roey, Saranda Ombashi, Irene M.J. Mathijssen, Aebele B. Mink van der Molen, Sarah L. Versnel

**Affiliations:** * Department of Plastic and Reconstructive Surgery and Hand Surgery, Erasmus Medical Center, Rotterdam; † European Reference Network for Rare and/or Complex Craniofacial Anomalies and Ear, Nose, and Throat Disorders, Pan-European, Virtual; ‡ Department of Pediatric Plastic and Reconstructive Surgery, University Medical Center, Utrecht, The Netherlands

**Keywords:** Cleft lip, cleft palate, dutch, normative values, PROM

## Abstract

Patient-Reported Outcome Measures (PROMs), such as the CLEFT-Q, have become essential for outcomes in patients with CL/P. Normative values of the CLEFT-Q for non-CL/P peers have not yet been established. This study aims to establish normative values for the CLEFT-Q in the general Dutch population. Dutch nationals aged 16-24 years without CL/P were recruited through an online survey. Participants completed the CLEFT-Q, excluding the lip scar and eating and drinking scales. Data were weighted based on the Dutch Central Bureau of Statistics. Normative values were calculated as means and standard deviations, stratified by sex and education category. Tobit regression models were used to analyze associations between CLEFT-Q scores and demographic variables. In total, 870 participants responded, of which 160 were excluded due to potential craniofacial anomalies. Significant variations in CLEFT-Q scores based on sex were found, with females scoring lower than males. Level of education had a modest impact on CLEFT-Q scores, with lower education having lower scores on certain scales. Age marginally influenced CLEFT-Q scores, with younger participants scoring lower than older participants. Positive correlations were found between all CLEFT-Q scales. The strongest correlation was observed between the social and school functioning scales. This study provides the first set of normative values for the CLEFT-Q in the Dutch general population. Significant differences in CLEFT-Q scores based on sex, level of education and age were found. These normative values are useful for clinicians interpreting CLEFT-Q scores and help make informed decisions.

A cleft lip and or palate (CL/P) is the most prevalent congenital craniofacial condition worldwide.^1^ Patients with CL/P require multidisciplinary care to improve breathing, speaking, eating, hearing and appearance.^2^ Historically, health outcomes of patients with CL/P were determined using clinical interpretation and clinical outcome measures. However, a more holistic view of cleft care, which includes the self-perception of patients, has gained favor.^3^ This interest in the patient’s perspective initiated many Patient-Reported Outcome Measures (PROMs) to be developed for patients with CL/P. PROMs allow for the quantification of self-perceived outcomes in different domains, such as health-related quality of life, patient satisfaction, symptoms, and functioning.^4^ Currently, PROMs are used complementarily to evaluate clinical outcomes of treatment. PROMs can be used to accurately collect data from pediatric patients aged 8 and above.^5^

One widely used PROM developed for patients with CL/P is the CLEFT-Q.^6–8^ The validity, internal responsiveness, and normative values of the CLEFT-Q have been studied and established for patients with CL/P;^6^ however, normative values based on the general or non-CL/P population have not. This lack of normative values for the general population causes the interpretation of the CLEFT-Q results and clinical decision-making to be more difficult. Normative population values give clinicians a better idea of which CLEFT-Q scores are normal at a given age and help determine if additional (surgical) intervention is advised, or if a different approach would be more useful for the patient. Therefore, the aim of the current study is to generate and report on the CLEFT-Q normative values for the general Dutch population, including specific values based on sex and education category.

## METHODS

### Study Population and Data Collection

In this cross-sectional study, Dutch nationals without CL/P, or any other craniofacial anomaly, aged between 16 and 24 years were included. Participants were recruited for an online survey by a third-party company in May through June of 2023 through email. Participants had previously enrolled with the third-party company to participate in online. Potential participants were selected based on their age, which they had previously provided. The survey included an explanation of the study in laymen terms and collected information on demographic characteristics, including sex, date of birth, level of education, area of residence (Nielsen district), and presence of any craniofacial anomaly treated by a medical doctor, as well as the actual CLEFT-Q scales. The survey without the CLEFT-Q scales can be found in the appendix, Supplemental Digital Content 2, http://links.lww.com/SCS/H64. Participants had to complete all CLEFT-Q scales, except for the lip scar and eating and drinking scales. The eating and drinking scale was excluded because it is a checklist and not a scale. The lip scar scale was excluded because this scale can only be answered in relation to having a repaired cleft lip. Participants received a small reimbursement (4.50 euro) for completion of all included CLEFT-Q scales. The data were collected and de-identified by the third-party company before statistical analysis.

### Questionnaire

The CLEFT-Q consists of twelve scales and one checklist designed to assess self-perception on the domains of appearance (face, jaw, nose, nostrils, teeth, and cleft lip scar), facial function (speech functioning, eating, and drinking), and health-related quality of life (speech distress, psychological, social, and school functioning). Each scale of the CLEFT-Q, except for the eating and drinking scale results in a score of 0 (lowest) to 100 (highest).

### Data Analysis and Statistics

The study population was stratified by sex (male versus female) and education (practical versus theoretical) according to the definitions by the Dutch Central Bureau of Statistics (CBS). Distributions were not representative of the general population. Hence, post-hoc weighting was applied, based on distributions reported by the CBS of 2022 of the Dutch population. Normative values were presented separately for men and women, with practical or theoretical education, as means and standard deviations.

Tobit regression models were fitted for each of the CLEFT-Q scales, to account for the censored nature of the PROM scores.^9^ In each model, sex, education, and age were included to investigate their relationship with the scores of each CLEFT-Q scale. As all participants were 16 years or older, age was included in the models by counting the years above 16 to estimate regression coefficients. Spearman correlations were calculated between scores of the CLEFT-Q scales. Correlations below 0.5, between 0.5 and 0.7, and above 0.7, were considered weak, moderate, and strong, respectively. R statistical software (v4.3.1) was used for statistical analysis and visualization, and a significance level of 0.05 was applied.

## RESULTS

### Participant Characteristics

A total of 870 individuals of Dutch nationality participated in the present study. Among these, 160 individuals were excluded due to a craniofacial anomaly for which treatment was provided by a medical doctor or because they chose not to disclose whether they had a craniofacial anomaly that was treated by a medical doctor. In the unweighted dataset, the mean age was 20.2 years (weighted mean 20.3 y). Of the participants, 39.0% were men (weighted 50.9%), and 69.7% had a theoretical education background (weighted 54.0%). The majority of participants originated from the western district (31.8%; weighted 29.3%), followed by the eastern district (22.5%; weighted 21.8%) and southern district (22.5%; weighted 22.5%), the Northern district (12.5%; weighted 10.4%) and the Randstad (10.5%; 16.0%). Individual participant weights ranged from 0.51 to 3.51. Detailed characteristics of the participants are provided in Supplemental Table 1, Supplemental Digital Content 1, http://links.lww.com/SCS/H63.

### Cleft-Q Outcomes

Supplemental Table 2, Supplemental Digital Content 1, http://links.lww.com/SCS/H63 and Supplemental Table 3, Supplemental Digital Content 1, http://links.lww.com/SCS/H63 provide parallel presentations of the normative values (mean and standard deviation) for each CLEFT-Q scale, stratified by both sex and education category, within the general population of the Netherlands. Within the overall sample, mean scores for the appearance scales ranged from 61.3 to 68.7, and from 60.2 to 77.7 for the health-related quality of life scales.

**FIGURE 1:**
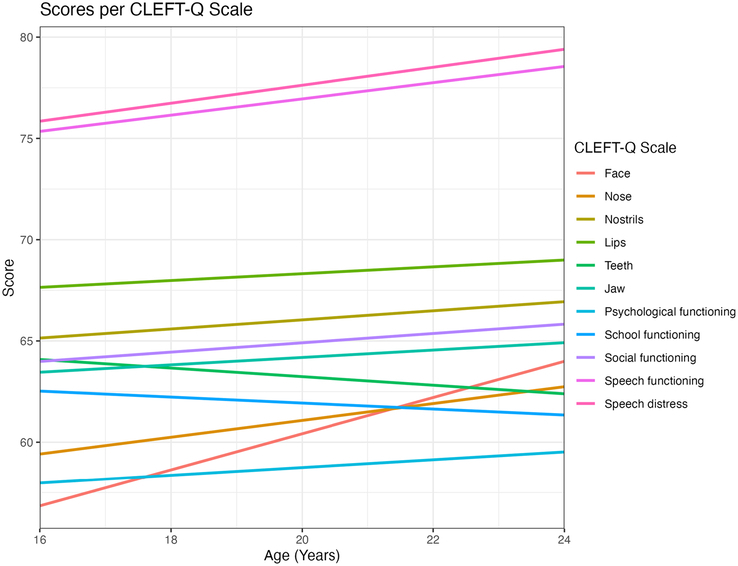
Mean score per CLEFT-Q scale for each age.

**FIGURE 2:**
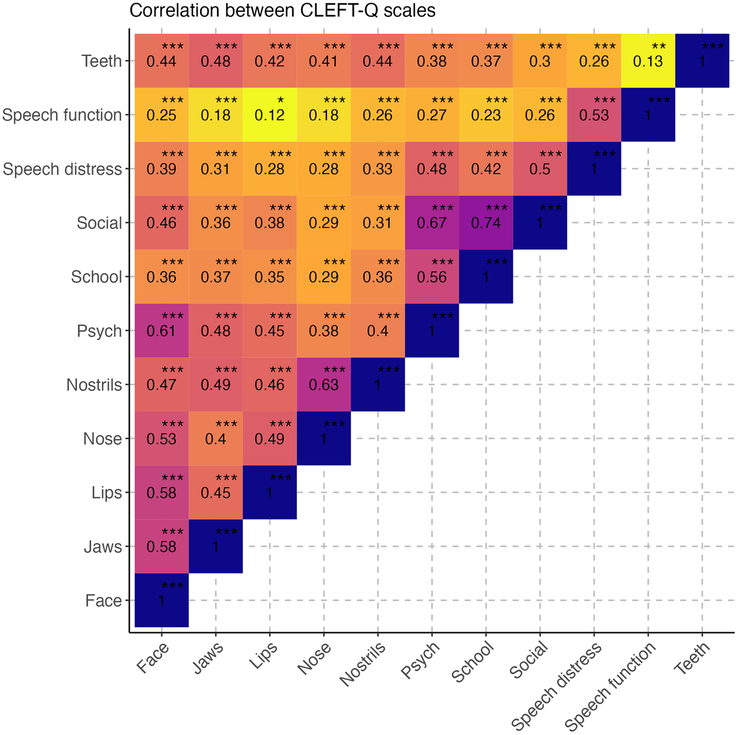
Heat map of correlations between CLEFT-Q scales.

Multivariable Tobit regression (Supplemental Table 4, Supplemental Digital Content 1, http://links.lww.com/SCS/H63) revealed significant associations between sex and appearance scales, with the exception of the CLEFT-Q teeth scale. On average, women attained lower scores on all the appearance scales in comparison to men. Similar associations were observed between sex and the psychological functioning scale (*P*=0.003) as well as the school-functioning scale (*P*<0.001). The effect of education category and age were less discernible in the current sample and did not reach statistical significance for most scales. The education levels seemed to have a minor impact on psychological functioning scale (*P*=0.014) and speech distress scale (*P*=0.040). Age only significantly impacted the face scale scores (*P*=0.001), with higher scores in older patients. The mean scores for all CLEFT-Q scales per age are visualized in Fig. 1. The overall effect of the residential area was not significant. Figure 2 shows the correlation between CLEFT-Q scales on a heat map. All correlations were positive. A strong correlation was seen between the CLEFT-Q social scale and the school-functioning scale (0.74).

## DISCUSSION

In the current study, normative values of the CLEFT-Q for the general population of the Netherlands are provided. The participants ranged from 16 years to 24 years. This range was chosen to correspond to the ages at which patients with CL/P are at the end of treatment. During this time period patients typically decide if additional surgery to the lips and/or nose to improve aesthetics is desired. Therefore, this is an important age group to generate non-CL/P peer normative values for. In addition, research by Ombashi et al^10^ showed that patients with CL/P aged 8 or 12 years more often completed the CLEFT-Q together with parents/caregivers than 15-year-old patients with CL/P. Parents reported more positively than patients. Thus, normative values from non-CL/P peers from 16 years up to 24 years, mitigate the influence of parents on the average scores.

The findings of this study highlight differences in scores between males and females within the appearance scales. These align with previous research into self-perceived appearance and sex. A study on self-perceived satisfaction of body appearance between men and women by Quittkat et al^11^ showed that women score lower on average compared with men. This study did not specifically assess facial appearance; however, similar differences seem to be present in the current study. A study by Paganini et al^12^ found that in patients with a unilateral cleft lip and palate, women had higher appearance-related anxiety than men as well. Regarding the psychosocial and school-functioning scales, scores were lower for women, although less pronounced than for the appearance scales. Research by Bleidorn et al^13^ also found women had lower average self-esteem than men and reported lower satisfaction relating to psychosocial outcomes.

When assessing the effect of age, statistical significance was not reached for any scale except for the facial appearance scale (*P*=0.001). This can be explained by the small age range included in the current study. However, previous research on this topic has found statistical differences in self-perception and esteem between ages, specifically between adolescents and middle-aged adult women.^13–16^ With adult women scoring significantly better. Nevertheless, these findings are not universally accepted, as other studies did not find statistical differences between age groups within women.^11^

Similar patterns for psychosocial functioning and education levels were found by Mitchell et al.^17^ Participants with lower levels of education reported lower psychosocial-functioning scores. The strong positive correlation found between the social-functioning and school-functioning scales in Fig. 2 underlines this finding. Similar positive correlation between the social scale and school functioning in 11 to 17-year-old with CL/P were found in a study by Apon et al^18^ (range: 0.78–-85). It was noted that a significant overlap existed between the items in the school-functioning scale and the items in the social scale.^18^ Thus, the strong positive correlation found in this study was to be expected.

Concerning the higher levels of speech distress in the lower educational levels this study found, previous research has reported that there is a higher prevalence of people of lower socioeconomic status (SES) in the lower educational levels. People of lower SES have been noted to have worse speech than peers of a higher SES.^19,20^ In addition, research by the CBS in the Netherlands showed that lower education levels in the Netherlands tend to have a higher prevalence of first or second-generation non-Dutch-speaking immigrants.^21^ This could be an additional factor influencing the higher levels of speech distress in the lower educational levels. Nevertheless, data on ethnicity were not collected in this study, so this hypothesis could not be tested.

### Comparison to Previous Research on CLEFT-Q

When comparing the outcomes of the current study to the normative values of the CLEFT-Q for individuals with CL/P generated by Klassen and colleagues similarities can be found. Participants with CL/P aged 16 to 17, 18 to 20, and older than 21 years have lower mean scores for most scales than young patients (8 y). Other literature supports this pattern, in a study by Balwin et al^16^ adults and children were reported to have greater self-esteem than adolescents and young adults. This same pattern can be seen in the study by Klassen et al^6^, where (young) adolescents tended to score lower than older age groups on average This pattern is also seen in research by van der Knaap-Kind, who found that patients with CL/P scores of the CLEFT-Q teeth scale, improved with age into adulthood.^22^ A study by Ombashi et al^23^ reported similar findings for CLEFT-Q scores, where young patients scored higher than adolescents, but adults also scored higher than adolescents for the CLEFT-Q jaw, lip, and scar scales. These patterns of improving scores with older patients scoring higher is noteworthy as the current study with non-CL/P peers adheres to this pattern too. Therefore, we propose that improvements in CLEFT-Q scores for patients with CL/P are not only the cause of treatments but of age as well.

**FIGURE 3:**
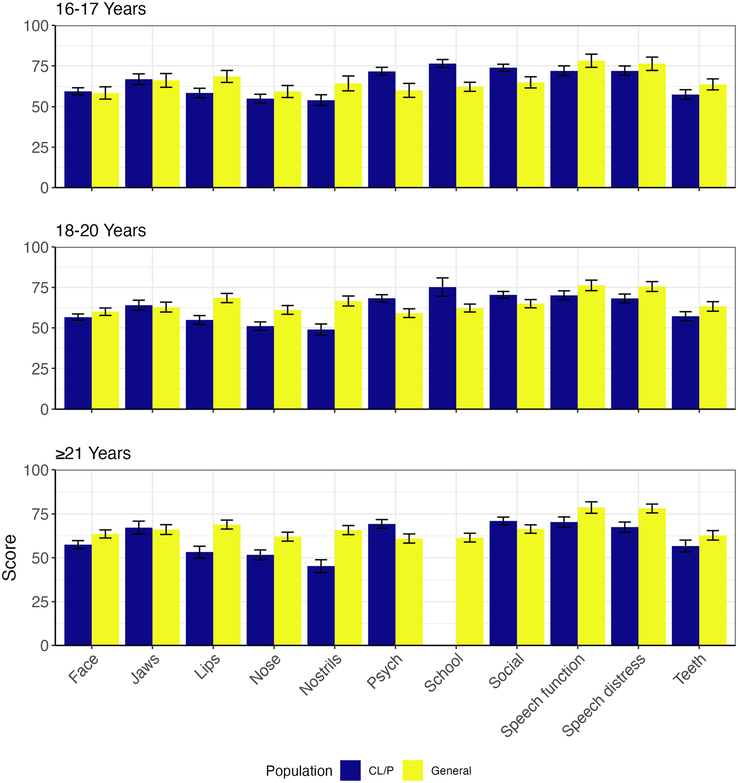
Mean scores per age group of current study versus mean scores of the study by Klassen et al.

Finally, patients with CL/P score lower than their non-CL/P peers on most scales. This is visualized in Fig. 3. Non-CL/P peers scored higher for the facial appearance, lips, nose, nostrils, speech function, and speech distress scales. Scores found for the appearance of the jaw scale were comparable. The study by Klassen and colleagues, reports higher mean scores for the CL/P population on psychosocial functioning, school life, and the social life scale.

We expect that the differences in the appearance scales are caused by the effects of the cleft lip. The malformation of the cleft lip and subsequent surgery influences not only the shape and symmetry of the lips but also the nose and thus the face. Nguyen and colleagues report that the most common deformities of the nose for patients with CL±P, were septal deviation, defects of the upper part of the nostril contour, narrow sill, and low position of the ala. Furthermore, many patients with CL/P require additional surgery to the lips and nose to improve aesthetics.^24^

Regarding the appearance of the teeth, we postulate that the lower mean score is mostly influenced by a cleft alveolus and possible oral health issues being more prevalent in patients with CL/P.^25^ It is, however, not possible to differentiate between cleft type and age using the data provided by the study of Klassen and colleagues, nor was clinical data on oral health collected for non-CL/P peers, therefore this hypothesis cannot be tested in the current study. There was no clear difference in mean scores for the CLEFT-Q appearance of the jaw scale.

The speech functioning and speech distress scales mean scores were higher for the non-CLP population for all age groups. The difference in mean scores of the speech function scale and distress scale can be attributed to the cleft palate being originally present in some of the phenotypes of CL/P. Even after surgery and speech therapy, speech can remain impeded due to remaining velopharyngeal insufficiencies and is often a source of dissatisfaction for patients with a cleft palate.^26^

For the psychosocial-functioning, school-functioning, and social-functioning scales, Klassen and colleagues report higher mean scores for patients with CL/P than we found in the current study for non-CL/P peers. This finding is not supported by previous literature, where patients with CL/P scored lower than non-CL/P peers for psychosocial functioning, and emotional and social functioning.^27^ We postulate that this difference could be caused by multiple factors. Firstly, data collection of the study by Klassen et al^6^ took place between October 2014 and November 2016. Mental health among Dutch adolescents and young adults has been worsening over the last years according to the Dutch Central Bureau of Statistics.^28^ Furthermore, studies on the topic of mental health in adolescents mentioned the COVID-19 pandemic as a catalyst for this increase in depression and anxiety.^29^ Research also suggests that depression and anxiety in the adult population dropped back to pre-COVID-19 pandemic levels, whereas levels in young adults did not.^30^ Anxiety and depression are noted to affect psychosocial functioning in research generated within the Netherlands Study of Depression and Anxiety (NESDA).^31^

Another explanation could be the use of social media. A systematic review by Keles et al^32^ concluded that, although the effect of social media use on depression, anxiety and psychological distress among adolescents is likely multifactorial, time spent on, activity of, investment in and addiction to social media is correlated to depression, anxiety, and psychological distress. The percentage of social media-using adults has grown from 5% in 2005 to 79% in 2019.^32^ The use of social media is, therefore, expected to have increased since the study by Klassen and colleagues compared with the current study. Furthermore, Ortiz-Ospina and colleagues reported that 18 to 24-year-old used social media most frequently of all age groups. The percentage of social media users for this group is nearly 100% in developed nations. In addition, time spent online by people aged 14 to 24 was reported to be the highest in the Netherlands (6.03 h/day).^32^ Therefore, the effects of social media could have on psychosocial functioning should not be underestimated. Finally, cultural differences could influence the average scores of the CLEFT-Q. In the study by Klassen and colleagues of the 2434 participants, 206 were Dutch (8.6%), and the other 2228 (91.4%) participants came from eleven other countries.

### Clinical Implications

The findings of the current study provide normative values for the CLEFT-Q of individuals without CL/P between 16 and 24-year-old in the Netherlands. These values represent the normative population benchmarks and identify patient characteristics that influence CLEFT-Q outcomes. For clinicians who are evaluating the CLEFT-Q results, it can be helpful to take these characteristics into consideration when assessing the scores of patients with CL/P. Furthermore, this information provides clinicians insight into the likelihood that a cleft-related issue is a primary cause if a patient with CL/P scores significantly lower than the normative population values. Thus, patients with a low CLEFT-Q score be assessed carefully, even if clinical outcomes are not noticeably lacking.

### Limitations

Firstly, this study was limited to the age of 16 and above. Consequently, investigating potential score variations throughout treatment from childhood to adulthood was impossible. Secondly, the nature of the data collection restricted the generalizability of the study sample’s representation to the Dutch population with internet access. However, this limitation was considered to have minimal influence on the results, given the target group. Thirdly, the CLEFT-Q has not been specifically developed for a non-CL/P population. However, validation of the CLEFT-Q for a non-CL/P population is not necessary to generate normative values. As there is no intention of using the CLEFT-Q routinely on non-CL/P peers it was deemed unnecessary to perform such a validation.

### Future Research

To gain deeper insights into the differences in self-reported satisfaction between individuals with CL/P and their non-CL/P counterparts, it is essential to examine and compare our study results with scores of Dutch patients with CL/P. This analysis should encompass individuals with CL/P at or near the end of treatment. In addition, it would be of interest to compare current CLEFT-Q scores to the scores of the research by Klassen and colleagues to see if average scores have changed. Finally, it would be useful to determine the minimal clinical important difference (MCID) for the CLEFT-Q. This would help clinicians by defining at what score patients are clinically significantly more dissatisfied compared with non-CL/P peers.

## CONCLUSION

This study provides the first normative population values of the CLEFT-Q for the Dutch population in late adolescence and early adulthood. Sex influences average scores of the CLEFT-Q, with female participants scoring lower on average. Younger participants had lower average scores, though less pronounced. Patterns found in the non-CL/P population follow trends found in previous research on the CLEFT-Q with patients with CL/P. In addition, CLEFT-Q scores may increase with age, regardless of treatment. Finally, level of education influenced average CLEFT-Q scores, with lower education levels having lower CLEFT-Q scores. All these findings facilitate the interpretation of the CLEFT-Q by clinicians and may help with (shared)decision-making.

## Supplementary Material

SUPPLEMENTARY MATERIAL

## References

[1] Salari N Darvishi N Heydari M . Global prevalence of cleft palate, cleft lip and cleft palate and lip: A comprehensive systematic review and meta-analysis. J Stomatol Oral Maxillofac Surg 2022;123:110–120 34033944 10.1016/j.jormas.2021.05.008

[2] Nederlandse vereniging plastisch chirurgen. Schisis - Richtlijn - Richtlijnendatabase. Accessed Sep 6, 2022. https://richtlijnendatabase.nl/richtlijn/behandeling_van_patienten_met_een_schisis/startpagina_schisis.html

[3] Sitzman TJ Allori AC Thorburn G . Measuring outcomes in cleft lip and palate treatment. Clin Plast Surg 2014;41:311–319 24607197 10.1016/j.cps.2013.12.001

[4] Patient-reported outcome measures: an overview. Accessed Sep 6, 2022.: https://www.researchgate.net/publication/50289838

[5] Bele S Chugh A Mohamed B . Patient-reported outcome measures in routine pediatric clinical care: a systematic review. Front Pediatr 2020;8:364. 32850521 10.3389/fped.2020.00364 PMC7399166

[6] Klassen AF Riff KWYW Longmire NM . Psychometric findings and normative values for the CLEFT-Q based on 2434 children and young adult patients with cleft lip and/or palate from 12 countries. CMAJ 2018;190:E455–E462. 29661814 10.1503/cmaj.170289 PMC5903887

[7] Wong Riff KWY Tsangaris E Goodacre TEE . What matters to patients with cleft lip and/or palate: an international qualitative study informing the development of the CLEFT-Q. Cleft Palate Craniofac J 2018;55:442–450. 29437508 10.1177/1055665617732854

[8] Riff KWYW Tsangaris E Goodacre T . International multiphase mixed methods study protocol to develop a cross-cultural patient-reported outcome instrument for children and young adults with cleft lip and/or palate (CLEFT-Q). BMJ Open 2017;7:e015467. 10.1136/bmjopen-2016-015467 PMC5253569 28077415

[9] Cunillera O Tobit Models. Encyclopedia of Quality of Life and Well-Being Research [Internet]. 2014 [cited 2023 Aug 21];6671–6. Available from: https://link.springer.com/referenceworkentry/10.1007/978-94-007-0753-5_3025

[10] Ombashi S Roey VL van Okkerse JME, Who Should Fill Out a Pediatric PROM? Psychometric Assessment From a Clinical Perspective in 567 Children With a Cleft https://doi.org/101177/27325016231209051 [Internet]. 2023 Nov 25 [cited 2024 Jan 24]; Available from: https://journals.sagepub.com/doi/10.1177/27325016231209051?icid=int.sj-full-text.citing-articles.5

[11] Quittkat HL Hartmann AS Düsing R . Body dissatisfaction, importance of appearance, and body appreciation in men and women over the lifespan. Front Psychiatry 2019;10:864. 31920737 10.3389/fpsyt.2019.00864 PMC6928134

[12] Paganini A Moss T Persson M . A gender perspective on appearance-related concerns and its manifestations among persons born with unilateral cleft lip and palate. Psychol Health Med 2021;26:771–778 32720821 10.1080/13548506.2020.1800055

[13] Bleidorn W Arslan RC Denissen JJA, . Personality processes and individual differences age and Gender Differences in Self-Esteem-A Cross-Cultural Window. 2015 [cited 2023 Sep 19]; Available from: 10.1037/pspp0000078.supp 26692356

[14] Tiggemann M McCourt A . Body appreciation in adult women: relationships with age and body satisfaction. Body Image 2013;10:624–627. 23954196 10.1016/j.bodyim.2013.07.003

[15] Öberg P Tornstam L . Body images among men and women of different ages. Ageing Soc 1999;19:629–644.

[16] Baldwin SA Hoffmann JP . The dynamics of self-esteem: a growth-curve analysis. J Youth Adolesc 2002;31:101–113.

[17] Mitchell UA Ailshire JA Brown LL . Education and psychosocial functioning among older adults: 4-year change in sense of control and hopelessness. J Gerontol B Psychol Sci Soc Sci 2018;73:849. 27013537 10.1093/geronb/gbw031 PMC6283311

[18] Apon I Van Leeuwen N Koudstaal MJ . Optimizing the psychosocial function measures in the international consortium for health outcomes measurement standard set for cleft. Plast Reconstr Surg 2023;151:274E–281E. 10.1097/PRS.0000000000009852 PMC9869941 36696325

[19] Spencer S Clegg J Stackhouse J . Contribution of spoken language and socio-economic background to adolescents’ educational achievement at age 16 years. Int J Lang Commun Disordnet 2017;52:184–196. 10.1111/1460-6984.12264 27432281

[20] Ariani MG Ghafournia N . The Relationship between socio-economic status, general language learning outcome, and beliefs about language learning. Int Educ Stud 2016;9:89–98

[21] Dourleijn en Jaco Dagevos E Vluchtelingengroepen in Nederland

[22] van der Knaap-Kind LS Ombashi S Van Roey V . Evaluation and recommendations of the oral health, oral function, and orofacial aesthetics-related measures of the ICHOM Standard Set for Cleft Lip and Palate. Int J Oral Maxillofac Surg 2024;53:563–570. 38228465 10.1016/j.ijom.2024.01.001

[23] Ombashi S Kurniawan MSIC Koudstaal MJ . Most efficient and meaningful patient-reported appearance assessment in different cleft types and age groups with CLEFT-Q. Plast Reconstr Surg 2024;153:120E–129E. 10.1097/PRS.0000000000010523 37054385

[24] Nguyen HL Nguyen VM Tran XP . Cleft lip/nasal deformities after plastic surgery for unilateral cleft lip/palate: a prospective study at a large hospital in Vietnam. Clin Cosmet Investig Dent 2021;13:305–314. 10.2147/CCIDE.S320636 PMC8292625 34295190

[25] Al-Dajani M . Comparison of dental caries prevalence in patients with cleft lip and/or palate and their sibling controls. Cleft Palate Craniofac J 2009;46:529–531. 20050376 10.1597/08-003.1

[26] Morén S Lindestad PÅ Stålhammar L . Speech in adults treated for unilateral cleft lip and palate as rated by naïve listeners, speech-language pathologists, and patients. J Plast Reconstr Aesthet Surg 2022;75:3804–3812. 36064511 10.1016/j.bjps.2022.06.026

[27] Hunt O Burden D Hepper P . Self-reports of psychosocial functioning among children and young adults with cleft lip and palate. Cleft Palate Craniofac J 2006;43:598–605. 16986986 10.1597/05-080

[28] Mental health has worsened among young people | CBS [Internet]. [cited 2024 Mar 12]. Available from: https://www.cbs.nl/en-gb/news/2022/22/mental-health-has-worsened-among-young-people

[29] Wang S Chen L Ran H . Depression and anxiety among children and adolescents pre and post COVID-19: a comparative meta-analysis. Front Psychiatry 2022;13:917552. 35990058 10.3389/fpsyt.2022.917552 PMC9381924

[30] Gruber J Hinshaw SP Clark LA . Young adult mental health beyond the COVID-19 era: can enlightened policy promote long-term change? Policy Insights Behav Brain Sci 2023;10:75. 36942264 10.1177/23727322221150199 PMC10018249

[31] Saris IMJ Aghajani M van der Werff SJA . Social functioning in patients with depressive and anxiety disorders. Acta Psychiatr Scand 2017;136:352–361 28767127 10.1111/acps.12774 PMC5601295

[32] Keles B McCrae N Grealish A . A systematic review: the influence of social media on depression, anxiety and psychological distress in adolescents. Int J Adolesc Youth 2020;25:79–93.

