# Supplementary material for: Normative CLEFT-Q Data From the General Dutch Population
Source: J Craniofac Surg. 2024 Nov 21;36(3):907–11. doi: 10.1097/SCS.0000000000010882 (PMC12020395; doi:10.1097/SCS.0000000000010882)
Supplement: SUPPLEMENTARY MATERIAL [file scs-36-0907-s001.docx]

| **Supplemental Table 1 – Participant characteristics** | | | |
| --- | --- | --- | --- |
|  |  | **Unweighted data** | **Weighted data** |
| Sex (%) | Male | 277 (39.0%) | 361 (50.9%) |
|  | Female | 433 (61.0%) | 349 (49.1%) |
|  |  |  |  |
| Age | Mean (SD) | 20.2 (2.54) | 20.3 (2.57) |
|  | Median [IQR] | 20.0 [18-22] | 20 [18-23] |
|  |  |  |  |
| Age category (%) | 16-17 years | 128 (18.0%) | 124 (17.4%) |
|  | 18-20 years | 247 (34.8%) | 244 (34.3%) |
|  | 21-24 years | 335 (47.2%) | 342 (48.3%) |
|  |  |  |  |
| Education (%) | Theoretical | 495 (69.7%) | 383 (54.0%) |
|  | Practical | 215 (30.3%) | 327 (46.0%) |
|  |  |  |  |
| Nielsen district (%) | Randstad (Amsterdam, Rotterdam, The Hague and suburbs) | 75 (10.5%) | 111 (16.0%) |
|  | West (Utrecht, Noord-Holland, Zuid-Holland) | 226 (31.8%) | 208 (29.3%) |
|  | North (Groningen, Friesland, Drenthe) | 89 (12.5%) | 74 (10.4%) |
|  | East (Overijssel, Gelderland, Flevoland) | 160 (22.5%) | 155 (21.8%) |
|  | South (Zeeland, Noord-Brabant, Limburg) | 160 (22.5%) | 160 (22.5%) |

| **Supplemental Table 2 - CLEFT-Q reference values for men and women in the general population of the Netherlands (weighted)** | | | | | | |
| --- | --- | --- | --- | --- | --- | --- |
|  | **Men** | | **Women** | | **All** | |
|  | **(N = 361)** | | **(N = 349)** | | **(N = 710)** | |
| **Scale** | **Mean** | **SD** | **Mean** | **SD** | **Mean** | **SD** |
| *Face* | 64.8 | 18.5 | 58.1 | 16.7 | 61.5 | 17.9 |
| *Jaw* | 68.3 | 21.6 | 61.5 | 23.1 | 65.0 | 22.6 |
| *Lips* | 70.2 | 20.2 | 67.2 | 20.7 | 68.7 | 20.5 |
| *Nose* | 63.0 | 18.6 | 59.5 | 21.6 | 61.3 | 20.2 |
| *Nostrils* | 66.6 | 21.1 | 65.0 | 22.9 | 65.8 | 22.0 |
| *Psychological functioning* | 64.9 | 21.8 | 55.3 | 18.3 | 60.2 | 20.7 |
| *School functioning* | 63.4 | 19.5 | 60.3 | 16.3 | 61.9 | 18.1 |
| *Social functioning* | 69.1 | 20.0 | 62.1 | 16.7 | 65.7 | 18.8 |
| *Speech distress* | 77.5 | 19.9 | 77.9 | 17.5 | 77.7 | 18.5 |
| *Speech functioning* | 77.5 | 21.0 | 76.2 | 18.9 | 76.9 | 20.0 |
| *Teeth* | 64.2 | 21.4 | 62.0 | 19.7 | 63.1 | 20.6 |

| **Supplemental Table 3 – CLEFT-Q reference values stratified by sex and education category? in the general population of the Netherlands** | | | | | | | | | | | | | | |
| --- | --- | --- | --- | --- | --- | --- | --- | --- | --- | --- | --- | --- | --- | --- |
|  | **Theoretical** | | | | | |  | **Practical** | | | | | |  |
|  | **Men** | | **Women** | | **All** | | | **Men** | | **Women** | | **All** | | |
|  | **(N = 170)** | | **(N = 213)** | | **(N = 383)** | | | **(N = 192)** | | **(N = 135)** | | **(N = 327)** | | |
|  | **Mean** | **SD** | **Mean** | **SD** | **Mean** | **SD** | | **Mean** | **SD** | **Mean** | **SD** | **Mean** | **SD** | |
| *Face* | 64.7 | 17.2 | 58.7 | 15.3 | 64.9 | 19.6 | | 57.1 | 18.6 | 64.7 | 17.2 | 58.7 | 15.3 | |
| *Jaw* | 70.3 | 21.9 | 61.4 | 22.5 | 66.6 | 21.4 | | 61.8 | 24.0 | 70.3 | 21.9 | 61.4 | 22.5 | |
| *Lips* | 71.0 | 18.2 | 67.4 | 20.0 | 69.5 | 21.8 | | 66.9 | 21.9 | 71.0 | 18.2 | 67.4 | 20.0 | |
| *Nose* | 63.1 | 17.9 | 60.0 | 21.4 | 62.9 | 19.3 | | 58.6 | 21.9 | 63.1 | 17.9 | 60.0 | 21.4 | |
| *Nostrils* | 68.3 | 20.7 | 65.7 | 21.8 | 65.1 | 21.4 | | 63.9 | 24.5 | 68.3 | 20.7 | 65.7 | 21.8 | |
| *Psychological functioning* | 63.5 | 20.9 | 55.8 | 17.2 | 66.2 | 22.5 | | 54.4 | 19.8 | 63.5 | 20.9 | 55.8 | 17.2 | |
| *School functioning* | 65.5 | 18.1 | 61.2 | 14.5 | 61.6 | 20.6 | | 59.0 | 18.9 | 65.5 | 18.1 | 61.2 | 14.5 | |
| *Social functioning* | 69.3 | 19.2 | 62.5 | 15.6 | 69.0 | 20.7 | | 61.4 | 18.4 | 69.3 | 19.2 | 62.5 | 15.6 | |
| *Speech distress* | 75.6 | 19.2 | 79.1 | 15.8 | 79.3 | 20.5 | | 76.0 | 19.8 | 75.6 | 19.2 | 79.1 | 15.8 | |
| *Speech functioning* | 78.3 | 20.4 | 77.3 | 18.0 | 76.9 | 21.6 | | 74.5 | 20.3 | 78.3 | 20.4 | 77.3 | 18.0 | |
| *Teeth* | 66.1 | 20.6 | 63.3 | 18.7 | 62.6 | 21.9 | | 59.8 | 21.2 | 66.1 | 20.6 | 63.3 | 18.7 | |

| **Supplemental Table 4 – Multivariable linear regression coefficients for CLEFT-Q scales** | | | | | | | | |
| --- | --- | --- | --- | --- | --- | --- | --- | --- |
| **Scale** | **Adjusted R^2^** | **Sex** | | **Education** | | **Age** | |  |
|  |  | **(Female)** | | **(Practical)** | |  |  |  |
|  |  | **β** | **ρ** | **β** | **ρ** | **β** | **ρ** |  |
| Face | 0.041 | -6.59 | **<0.001** | 0 | 0.999 | 0.89 | **<0.001** |  |
| Nose | 0.004 | -3.67 | **0.037** | -0.04 | 0.983 | 0.46 | 0.170 |  |
| Nostrils | -0.003 | -2.18 | 0.293 | -1.58 | 0.471 | 0.30 | 0.449 |  |
| Lips | 0.002 | -4.64 | **0.015** | -0.47 | 0.817 | 0.24 | 0.502 |  |
| Teeth | 0.002 | -3.26 | 0.061 | -3.58 | 0.052 | -0.23 | 0.486 |  |
| Jaw | 0.019 | -8.62 | **<0.001** | -1.21 | 0.587 | 0.07 | 0.865 |  |
| Psychological functioning | 0.045 | -10.2 | **<0.001** | -0.12 | 0.946 | 0.11 | 0.722 |  |
| School functioning | 0.015 | -4.40 | **0.002** | -3.60 | **0.015** | -0.18 | 0.497 |  |
| Social functioning | 0.032 | -8.28 | **<0.001** | -0.96 | 0.554 | 0.16 | 0.577 |  |
| Speech functioning | -0.002 | 1.20 | 0.597 | 0.60 | 0.799 | 0.80 | 0.058 |  |
| Speech distress | -0.003 | -2.00 | 0.329 | -1.59 | 0.463 | 0.47 | 0.227 |  |
|  |  |  |  |  |  |  |  |  |
