# Supplementary material for: Normative CLEFT-Q Data From the General Dutch Population
Source: J Craniofac Surg. 2024 Nov 21;36(3):907–11. doi: 10.1097/SCS.0000000000010882 (PMC12020395; doi:10.1097/SCS.0000000000010882)
Supplement: SUPPLEMENTARY MATERIAL [file scs-36-0907-s002.docx]

# Appendix Normative values cleft-Q JCS

*This appendix includes the information on the study as provided to the participants, as well as the patient characteristics items. The first part is the original document in Dutch, the second part is the English translation.*

Beste …,

We willen je vragen om deel te nemen aan een onderzoek naar **hoe jij denkt over je uiterlijk**. In het Erasmus Medisch Centrum in Rotterdam behandelen we kinderen uit heel Nederland met een aangeboren aandoening van aangezicht. We willen beter begrijpen hoe hun zelfbeeld verschilt van dat van leeftijdsgenoten zonder aandoening van het aangezicht. Met dit onderzoek gaan we de uitkomsten van deze vragenlijsten vergelijken met de uitkomsten van vragenlijsten ingevuld door kinderen met een aandoening van het aangezicht. Jouw antwoorden zijn anoniem, dus nooit herleidbaar tot jou als persoon.

- **Wat is je geboortedatum?**
  - DD-MM-JJJJ
- **Wat is je geslacht?**
  - Man
  - Vrouw
  - Anders
- **Wat is de hoogste opleiding die je hebt gevolgd?**

*(Je hoeft hier geen diploma voor behaald te hebben)*

- - Master (WO of HBO)/postdoctoraal: hieronder valt ook doctoraal
  - Bachelor (WO of HBO): hieronder valt ook: hbo, hts, heao, kandidaatsopleiding
  - Propedeuse (WO of HBO)
  - Havo/VWO bovenbouw: hieronder valt ook: mms, hbs, gymnasium, lyceum, atheneum
  - Havo/VWO onderbouw: hieronder valt ook: mms, hbs, gymnasium, lyceum, atheneum
  - MBO-2/MBO-3/ MBO-4 /MBO voor 1998: hieronder valt ook: mts, meao, mhno, inas, mis, e.d.
  - VMBO (theoretisch/ gemengd): hieronder valt ook: mavo, ulo, mulo, ivo, vglo
  - VMBO (beroepsgericht)/MBO-1 (assistentenopleiding): hieronder valt ook: lts, ito, leao, lhno, huishoudschool, lavo, e.d.
  - Basisschool: hieronder valt ook: lagere school
  - Ik heb geen opleiding gevolgd
- **Waar ben je woonachtig?**
  - Dorp/Stad
    - Antwoord
  - Provincie
    - Noord-Holland
    - Zuid-Holland
    - Utrecht
    - Noord-Brabant
    - Zeeland
    - Limburg
    - Overijssel
    - Drenthe
    - Friesland
    - Groningen
    - Flevoland
    - Gelderland
- **Ben je geboren met een afwijking van je aangezicht waarvoor je onder behandeling bent (geweest) bij een medisch specialist?**

*(Voorbeeld: Afwijkende schedelvorm, aangeboren moedervlek)*

- Ja
- Nee

Dear …,

We would like to ask you to participate in a study on **what you think about your appearance.** In the Erasmus Medical Center in Rotterdam we treat children from all over the Netherlands with congenital conditions of the face. We would like to better understand how their self-image differs from peers without these facial conditions. In this study we want to compare the outcomes of several questionnaires answered by you with the answers of your peers with a facial conditions. Your answers will be anonymous, therefore never traceable to you as an individual.

- **What is your date of birth?**
  - DD-MM-YYYY
- **What is your sex?**
  - Male
  - Female
  - Other
- **What is the highest level of education you attended?**

*(You do not need to have obtained your diploma )*

- - Master (WO of HBO)/postdoctoral: This includes doctoral
  - Bachelor (WO of HBO): This includes: hbo, hts, heao, kandidaatsopleiding
  - Propedeuse (WO of HBO)
  - Havo/VWO secondary This includes: mms, hbs, gymnasium, lyceum, atheneum
  - Havo/VWO primary: hieronder valt ook: mms, hbs, gymnasium, lyceum, atheneum
  - MBO-2/MBO-3/ MBO-4 /MBO prior to 1998:This includes: mts, meao, mhno, inas, mis, e.d.
  - VMBO (theoretisch/ gemengd): This includs: mavo, ulo, mulo, ivo, vglo
  - VMBO (beroepsgericht)/MBO-1 (assistentenopleiding): This includes: lts, ito, leao, lhno, huishoudschool, lavo, e.d.
  - Primary school: this includes: lower school
  - I did not attend any education
- **Where do you currently live?**
  - Name of town/city
    - Answer
  - Province
    - North-Holland
    - South-Holland
    - Utrecht
    - North-Brabant
    - Zeeland
    - Limburg
    - Overijssel
    - Drenthe
    - Friesland
    - Groningen
    - Flevoland
    - Gelderland
- **Were you born with a congenital malformation of the face for which you have received treatment or are currently under treatment for at a medical specialist?**

*(Example: Malformation of the skull, congenital birthmark)*

- Yes
- No
